# Intraoperative methylene blue administration and postoperative delirium: a meta-analysis of surgical patients

**DOI:** 10.1007/s11845-026-04440-w

**Published:** 2026-05-21

**Authors:** Seçil Taylan, Gülden Küçükakça Çelik, Derya Gezer

**Affiliations:** 1 Kumluca Faculty of Health Sciences, Department of Surgical Diseases Nursing, Akdeniz University, Antalya, Turkey; 2 Department of Nursing, Nevsehir Haci Bektas Veli University Semra and Vefa Küçük Faculty of Health Sciences, Nevşehir, Turkey; 3 Department of Nursing, Faculty of Health Sciences, Tarsus University, Mersin, Turkey

**Keywords:** Critical care nursing, Intraoperative care, Meta-analysis, Methylene blue, Postoperative delirium

## Abstract

**Background:**

This meta-analysis aimed to determine the effect of intraoperative intravenous methylene blue administration on the incidence of postoperative delirium in adult surgical patients.

**Methods:**

This systematic review and meta-analysis was reported in accordance with PRISMA guidelines and registered in PROSPERO. A comprehensive search of seven electronic databases was performed from inception to January 2026 to identify randomized controlled trials evaluating intraoperative intravenous methylene blue administration and postoperative delirium in adult surgical populations. Study selection, data extraction, and methodological quality assessment were independently conducted by two reviewers using predefined eligibility criteria. Risk of bias was assessed using the Cochrane Risk of Bias 2 tool. Pooled odds ratios with 95% confidence intervals were calculated using a fixed-effect model due to the absence of between-study heterogeneity. Publication bias was evaluated using funnel plot inspection, Egger’s regression test, and Kendall’s tau test.

**Results:**

Three randomized controlled trials involving 779 patients were included. Intraoperative methylene blue administration was associated with a statistically significant reduction in postoperative delirium (OR = 0.35; 95% CI: 0.23–0.53; I^2^ = 0%).

**Conclusions:**

Intraoperative methylene blue administration was associated with a reduction in postoperative delirium in surgical patients. However, given the limited number of included studies, further large-scale randomized controlled trials are required.

## Introduction

Postoperative delirium (POD) is a common and clinically significant complication that typically emerges in the early postoperative period and is associated with a wide range of adverse outcomes [1]. Evidence consistently indicates that POD contributes to prolonged hospital length of stay, delayed functional recovery, and increased care dependency [2]. Importantly, its consequences are not limited to the immediate postoperative phase; POD may also lead to long-term cognitive decline, increased rehabilitation needs, and institutionalization [3]. Given these multidimensional impacts, early recognition, monitoring, and especially prevention of POD require a coordinated, multidisciplinary approach involving surgeons, anesthesiologists, and nurses, with the ultimate goal of improving patient-centered outcomes such as quality of life and satisfaction [4].

Although several predisposing and precipitating factors, including advanced age, comorbidities, lower educational level, surgical stress, and postoperative pain have been associated with POD, its underlying pathophysiology remains incompletely understood [5]. Current evidence, however, converges on neuroinflammation and oxidative stress as central mechanisms linking surgical and anesthetic exposure to perioperative neurocognitive disorders [6]. In particular, elevated levels of proinflammatory cytokines, such as IL-6 and IL-8, in both cerebrospinal fluid and systemic circulation have been consistently associated with the development of POD, suggesting a biologically plausible inflammatory pathway [7].

In this context, meta-analyses have played a critical role in consolidating the growing body of evidence on POD by providing more precise estimates of epidemiological burden and intervention effects [8–10]. However, the existing meta-analytic literature is largely fragmented, focusing on distinct but interconnected domains. For example, some meta-analyses have quantified the incidence and prevalence of POD [11], while others have examined patient-related and clinical risk factors, including frailty and inflammatory markers [12–15]. Additional syntheses have explored the influence of anesthetic techniques and agents, such as remimazolam and propofol [2], as well as intraoperative neurophysiological monitoring strategies, including electroencephalography (EEG)-guided anesthesia and burst suppression patterns [16, 17]. Furthermore, the downstream consequences of POD, particularly in elderly surgical populations, have also been systematically evaluated [18].

Despite this substantial body of synthesized evidence, a critical gap remains: most meta-analyses have focused on risk stratification, monitoring, or anesthetic management, whereas relatively less attention has been given to targeted pharmacological strategies addressing the underlying pathophysiological mechanisms, particularly neuroinflammation and oxidative stress. This gap highlights the need for evaluating interventions with neuroprotective properties that may modify the biological pathways implicated in POD development.

Methylene blue, an FDA-approved agent with a long history of clinical use, has recently attracted attention due to its neuroprotective, antioxidant, and anti-inflammatory properties. Preclinical and clinical evidence suggests that methylene blue may mitigate neuropathological and behavioral impairments in conditions such as Alzheimer’s disease [19], Parkinson’s disease [20], and traumatic brain injury [21]. Notably, experimental studies have demonstrated that methylene blue can reduce neuroinflammation and preserve blood–brain barrier integrity, mechanisms that are directly relevant to the pathogenesis of POD [22].

Given the central role of neuroinflammation in POD and the emerging neuroprotective profile of methylene blue, systematically synthesizing the available evidence on its intraoperative use represents a logical and clinically meaningful step. To the best of the authors’ knowledge, no meta-analysis has specifically evaluated the effect of methylene blue on postoperative delirium. Therefore, this study aims to systematically assess the impact of intraoperative methylene blue administration on the incidence of postoperative delirium in surgical patients based on the current evidence.

## Methods

This review was conducted in accordance with the Preferred Reporting Items for Systematic Reviews and Meta-Analyses (PRISMA) statement [9] and was registered in the PROSPERO database under the registration number CRD420261277123.

### Search strategy

As part of the search strategy, search terms were developed based on MeSH terminology as follows: AB (“methylene blue” OR “methylthioninium chloride”) AND AB (“postoperative delirium” OR “post-operative delirium” OR “postoperative cognitive disorder*” OR “POD”) AND AB (“randomized controlled trials” OR “RCT” OR “randomized controlled trials”). Using these search terms, the following databases were systematically searched without date restrictions up to January 2026: ScienceDirect, MEDLINE, Complementary Index, Academic Search Ultimate and Springer Nature, PubMed, and Web of Science (WoS).

### Inclusion and exclusion criteria

The research question of this meta-analysis was formulated using the Participant, Intervention, Comparator, and Outcome (PICO) framework [23]. The participants (P) consisted of adult individuals who underwent surgical procedures and were monitored during the postoperative period. The intervention (I) comprised methylene blue administered as a continuous intravenous infusion during the intraoperative period following anesthesia induction. The comparator (C) was defined as standard care or placebo without methylene blue administration. The outcome (O) of interest was postoperative delirium. Based on this PICO framework, the research questions were formulated as follows: *In surgical patients, does intravenously administered methylene blue during the intraoperative period following anesthesia induction reduce the incidence of postoperative delirium, and what is the magnitude of this effect?*

In the meta-analysis, studies published in languages other than English, publications reported only as abstracts or poster presentations, studies without a control group or those that did not assess delirium, studies published in non–peer-reviewed journals, as well as theses, letters to the editor, committee reports, conference proceedings, short reports, and expert opinions were excluded.

### Study selection

EndNote X8 reference management software was used to manage records retrieved from the literature search, and a three-stage screening process was applied. Each stage of the process was conducted independently by the authors (GKÇ and ST), followed by a joint discussion to reach consensus. In the first stage, only the titles of the identified studies were screened, and studies with uncertain eligibility were carried forward to the second stage. In the second stage, the abstracts of studies that passed the initial screening were evaluated. In the third and final stage, the full texts of the remaining studies were reviewed in detail according to the predefined inclusion criteria, and eligible studies were included in the meta-analysis.

### Data extraction

**Fig. 1:**
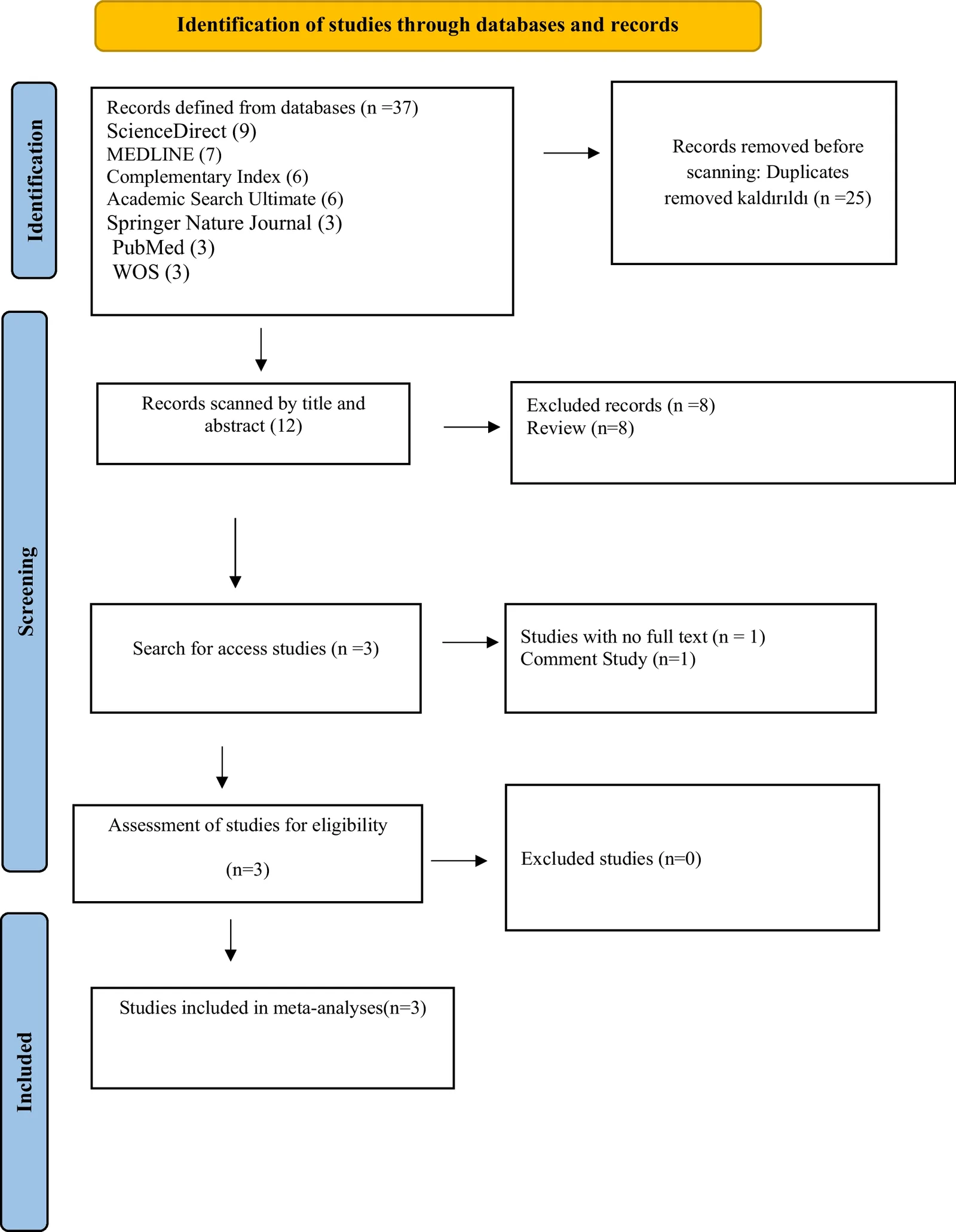
PRISMA flow diagram

Data extraction was performed using a standardized data extraction form that had been pilot-tested in advance. Using this form, the following information was systematically extracted from each study: author names, year of publication, country where the study was conducted, patient characteristics, type of surgery, and sample size, as well as the timing, dose, and route of administration of methylene blue. In addition, details regarding the delirium assessment tools used, delirium incidence, assessment time points and intervals, other measured outcomes, characteristics of the interventions applied, and information related to the comparator groups were also recorded (Fig. 1).

### Quality assessment

**Fig. 2:**
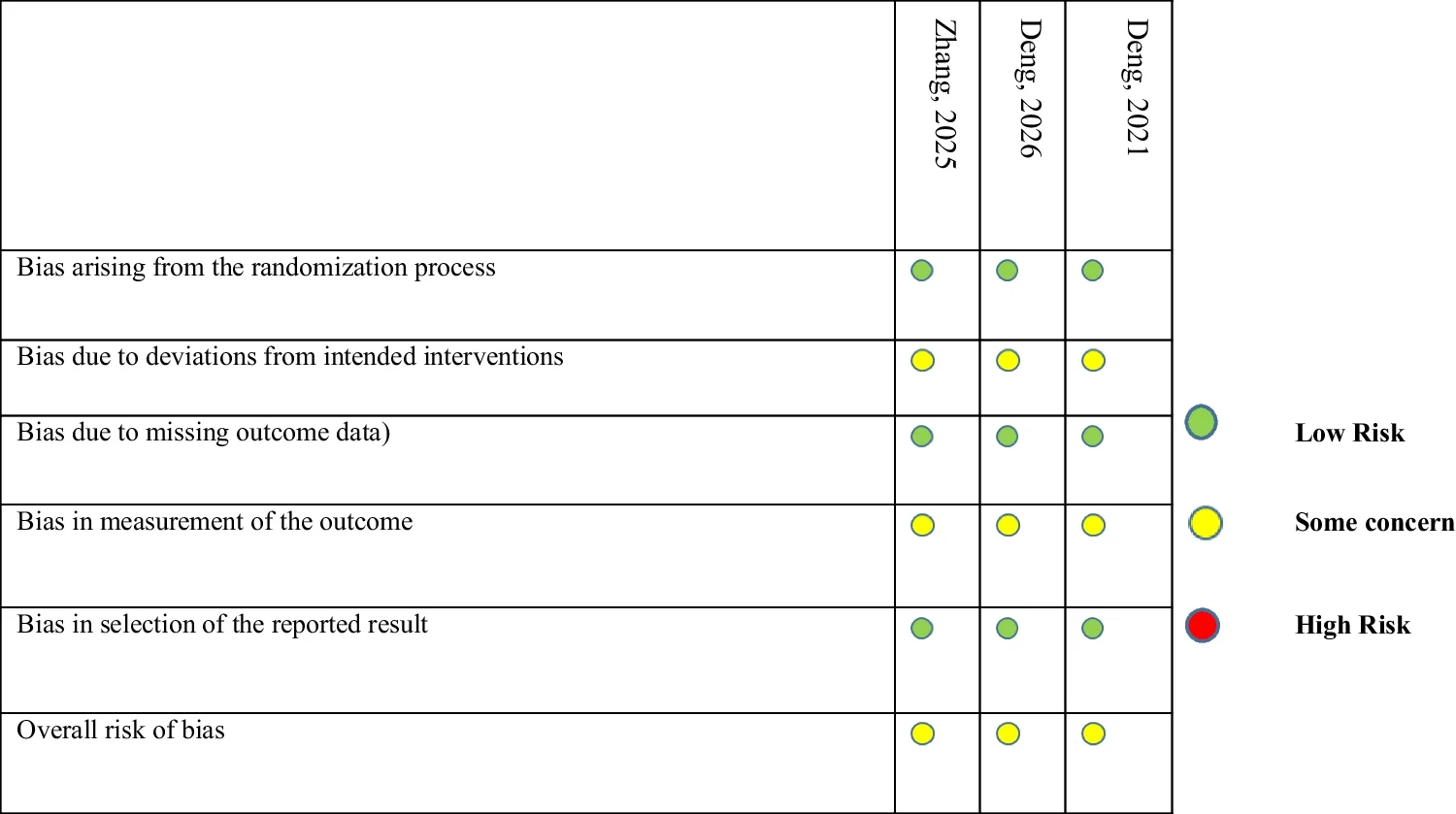
Risk of bias summary (ROB2)

Two authors (GKÇ and ST) independently assessed the methodological quality of the included studies based on the risk of bias and subsequently reached consensus through discussion. As all studies included in the meta-analysis were randomized controlled trials, the Cochrane Risk of Bias Tool (RoB 2) was used for quality assessment [24, 25] (Fig. 2).

### Ethical considerations

As this is a meta-analysis, it was not subject to an ethical appraisal process.

### Data analysis

As postoperative delirium was reported as a dichotomous outcome (presence/absence) in the included studies, effect sizes were calculated as odds ratios (ORs) and reported with 95% confidence intervals (CIs). Between-study heterogeneity was assessed using Cochran’s Q test, the I^2^ statistic, and τ^2^ (tau-squared). No significant heterogeneity was detected (I^2^ = 0%); therefore, a fixed-effect model was applied in the meta-analysis. The statistical significance of the pooled effect of methylene blue on postoperative delirium incidence was evaluated using the Z test. Publication bias was assessed through visual inspection of funnel plots, as well as Egger’s regression test and Kendall’s tau test; for Kendall’s tau, results were reported both with and without continuity correction. All statistical analyses were conducted using Comprehensive Meta-Analysis (CMA) software, and a *p* value < 0.05 was considered statistically significant.

## Results

### Search outcome

A total of 37 original records were identified from seven electronic databases during the literature search for the meta-analysis. Duplicates were automatically detected using EndNote reference management software, and 25 records were removed. In the first screening stage, the titles and abstracts of the remaining 12 studies were reviewed, and eight studies were excluded because they were review articles. The full texts of the remaining four articles were assessed for eligibility; one study was excluded as it was a commentary and did not meet the inclusion criteria. Ultimately, three studies met the inclusion criteria and were included in the meta-analysis.

### Quality assessment

The risk of bias of the randomized controlled trials included in the meta-analysis was assessed using the Cochrane Risk of Bias 2 (RoB 2) tool. All studies were at low risk of bias with respect to the randomization process, missing outcome data, and selection of the reported results. In the domains of deviations from intended interventions and measurement of the outcome, some concerns were identified due to the change in urine color associated with methylene blue and the study designs: two studies were single-blind [26, 27], and one study was open-label [28]. No study was judged to be at high risk of bias in any domain. When all domains were considered together, the overall risk of bias was rated as “some concerns” (Fig. 2).

### Characteristics of the included studies

A total of three studies were included in the meta-analysis, all of which were conducted in China. The study populations consisted of adults undergoing pancreatic surgery [26], elderly patients undergoing non-cardiac surgery [28], and elderly patients undergoing total knee and total hip arthroplasty [27].

In all studies, methylene blue was administered intravenously during the intraoperative period following anesthesia induction. Two studies administered methylene blue at a dose of 2 mg/kg over 60 min [27, 28]. In one study, an initial dose of 2 mg/kg was administered over 60 min, followed by an additional dose of 1 mg/kg approximately 30 min before the end of surgery [26].

Postoperative delirium was assessed using the 3D-Confusion Assessment Method (3D-CAM) [28] and the Confusion Assessment Method (CAM) [26, 27]. Delirium severity was evaluated using the Memorial Delirium Assessment Scale (MDAS) in one study [28] and the Delirium Rating Scale–Revised-98 (DRS-R-98) in another study [27], while severity was not assessed in one study [26]. All studies used the Richmond Agitation–Sedation Scale (RASS) to exclude patients with deep sedation (RASS ≤ − 3).

Adverse events and length of hospital stay were evaluated in all three studies. One study reported postoperative fever rates of 16.5% in the methylene blue group and 7.4% in the control group, with no significant differences reported for other adverse events [27]. The remaining studies reported no significant differences in adverse events between groups [26, 28]. Length of hospital stay was reported quantitatively in one study [27], while the other studies reported no significant differences between groups [26, 28].

Pain was assessed using the Numeric Rating Scale (NRS) in one study [26] and the Visual Analog Scale (VAS) in another study [27]. Postoperative cognitive impairment was assessed as postoperative cognitive dysfunction (POCD) in one study [26] and using the Telephone Interview for Cognitive Status–modified (TICS-m) in another study [27]. Additional outcomes included inflammatory biomarkers (IL-6, IL-8) [26], oxidative stress markers (superoxide dismutase and homocysteine) [28], and early level of consciousness assessed by the Coma Recovery Scale–Revised (CRS-R) [27].

### Effect of intraoperative methylene blue administration on postoperative delirium

Three randomized controlled trials including a total of 779 participants (methylene blue: *n* = 389; control: *n* = 390) were included in the analysis. Postoperative delirium was analyzed as a dichotomous outcome using odds ratios (OR).

**Fig. 3:**
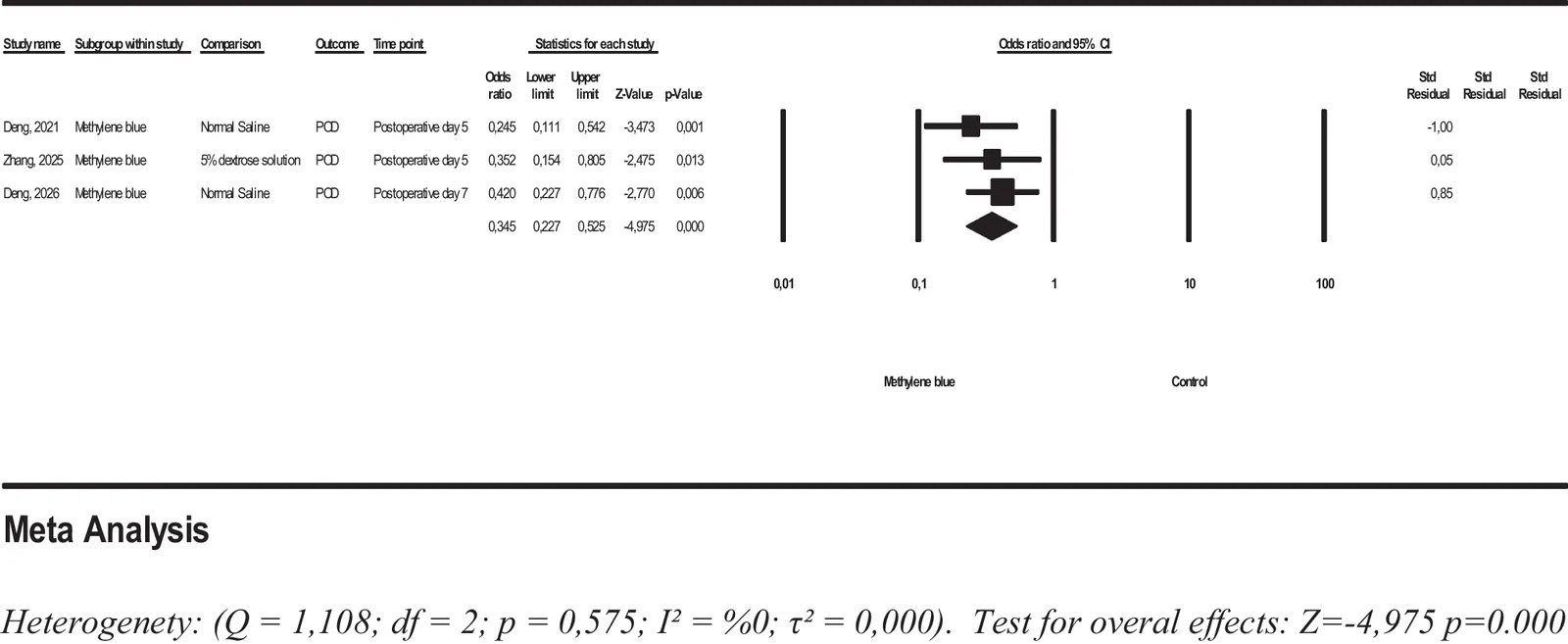
Forest plot showing the effect of methylene blue on postoperative delirium

Between-study heterogeneity was low (I^2^ = 0%), and a fixed-effect model was applied. The pooled analysis showed a statistically significant reduction in postoperative delirium associated with intraoperative methylene blue administration (OR = 0.35; 95% CI: 0.23–0.53; Z = − 4.98; *p* < 0.001) (Fig. 3).

Heterogeneity analysis showed Q = 1.11 (df = 2; *p* = 0.575; I^2^ = 0%; τ^2^ = 0.00).

**Fig. 4:**
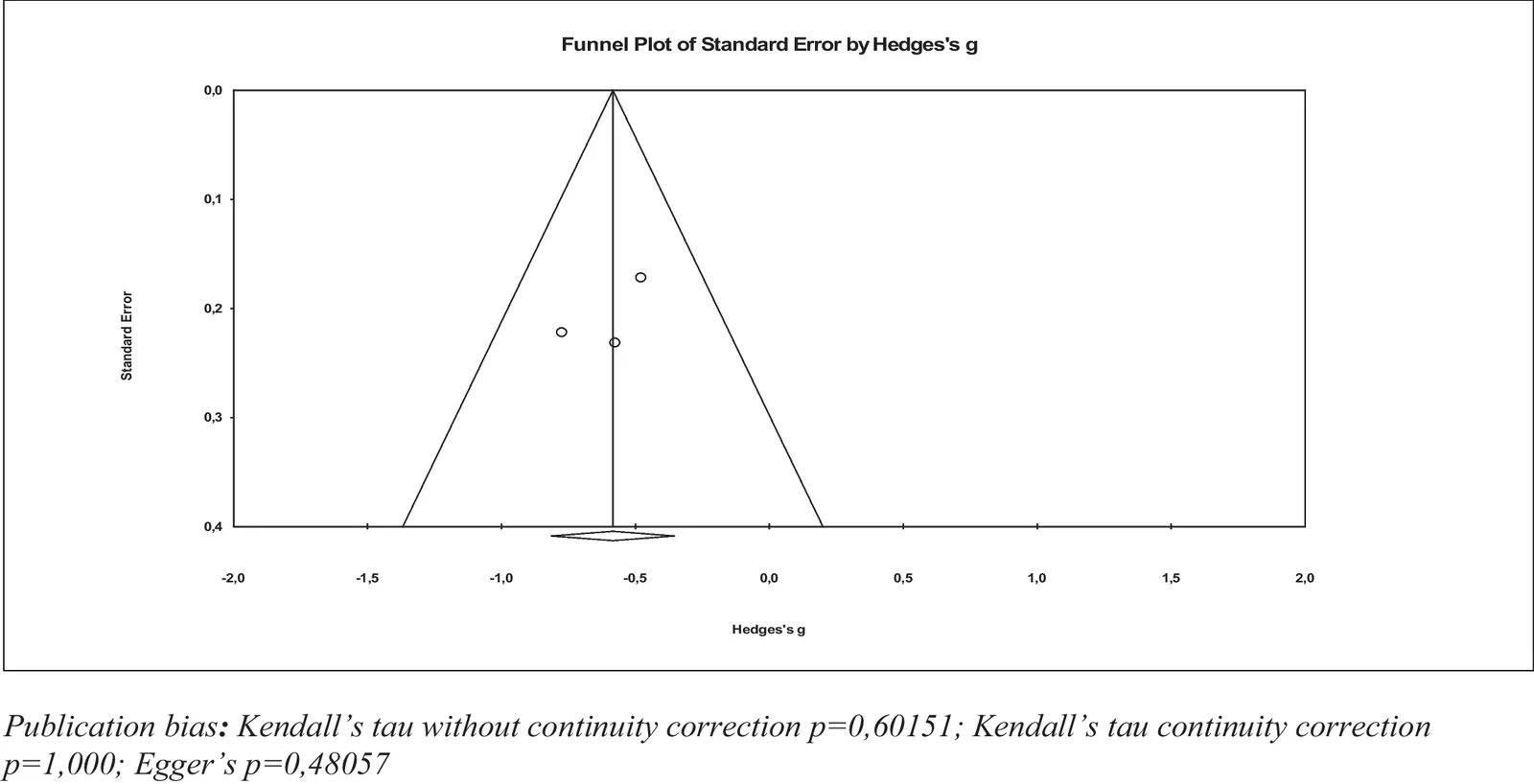
Funnel plot assessing publication bias for the effect of methylene blue on postoperative delirium

Publication bias assessment showed no apparent asymmetry in the funnel plot (Fig. 4). Kendall’s tau test was non-significant both without continuity correction (*p* = 0.6015) and with continuity correction (*p* = 1.000). The Egger regression test was also non-significant (*p* = 0.4806).

Subgroup analyses were not performed due to the limited number of included studies (*n* = 3) (Table 1).

**Table 1:** Characteristics of reviewed studies

1st author year country	Participant		Sample Size	Delirium measurement	Intervention	Frequency of ıntervention	Assessment ınterval	Assessment delirium	Conclussion
	Age	Type of surgery	Design	Other measurement					
Deng et al. [28] China	Median 67–69 years (IQR approx. 64–72)	Elderly patients (60–80 years), undergoing major non-cardiac surgery (thoracic, orthopedic, urologic, major abdominal)	Total *n* = 248 (MB group *n* = 124; Control *n* = 124)	3D-CAM (primary); MDAS for severity; RASS used to determine eligibility for assessment	Methylene blue 2 mg/kg IV infusion within 60 min after anesthetic induction	Single intraoperative dose	POD: twice daily for 5 postoperative days; POCD: postoperative day 7 and month 3 (TICS-m)	PACU delirium (30 min & 1 h post-extubation) + POD during postoperative days 1–5	Methylene blue significantly reduced the incidence of POD and early POCD without increasing serious adverse events
			Randomized, open-labelclinical trial	POCD assessed via comprehensive neuropsychological battery; plasma SOD and homocysteine; adverse events					
Deng et al. [26] China	Adults aged 55–80 years scheduled for elective pancreatic surgery (ASA I–III)	Pancreatic surgery (pancreaticoduodenectomy, distal pancreatectomy, total pancreatectomy, others)	Total *n* = 314 (Methylene blue *n* = 157; Control *n* = 157)	Confusion Assessment Method (CAM); assessed twice daily for 7 postoperative days; RASS used to determine eligibility	Intravenous methylene blue: 2 mg/kg within 60 min after anesthetic induction + 1 mg/kg within 30 min before end of surgery	Two intraoperative infusions (single perioperative course)	Delirium assessed twice daily with ≥ 6-h interval for up to 7 postoperative days or until discharge	POD diagnosed if CAM positive at any postoperative assessment	Intraoperative methylene blue significantly reduced the incidence of postoperative delirium without increasing adverse events
			Randomized, single-blind clinical trial	Plasma IL-6 and IL-8 levels; perioperative adverse events; immunovascular interaction assays (PBMC–endothelial adhesion); NRS pain scores					
Zhang et al. [27] China	Elderly patients Median 72.0 years (IQR 70.0–76.0 in control; 69.0–77.0 in MB group)	Joint replacement surgery (Total Knee Arthroplasty – TKA; Total Hip Arthroplasty – THA)	Total *n* = 217 (MB group *n* = 109; Control group *n* = 108)	Confusion Assessment Method (CAM); severity and subtype assessed with Delirium Rating Scale–Revised-98 (DRS-R-98)	Intravenous methylene blue 2 mg/kg diluted in 50 mL 5% glucose, infused within 60 min after anesthesia induction	Single intraoperative continuous infusion	Delirium assessed twice daily (morning & afternoon) with ≥ 6-h interval for 5 postoperative days	POD defined as any positive CAM assessment during postoperative days 1–5	Intraoperative intravenous methylene blue significantly reduced the incidence of postoperative delirium during hospitalization without increasing serious adverse events
			Randomized, single-blind controlled trial	CRS-R at 10 and 20 min post-extubation; VAS pain scores (postop days 0–5); TICS-m at 1 month; length of stay; adverse events					

## Discussion

In this meta-analysis, the effect of intraoperative methylene blue administration on postoperative delirium in surgical patients was evaluated by synthesizing the available evidence. To the best of the authors’ knowledge, this study represents the first meta-analysis to systematically assess the effects of intraoperatively administered methylene blue on postoperative delirium based on pooled evidence.

The pooled analysis demonstrated that intraoperative administration of methylene blue was associated with a statistically significant reduction in the incidence of postoperative delirium. No between-study heterogeneity was detected (I^2^ = 0%), suggesting that the direction of the effect was consistent across the included studies [8, 29]. In addition, no evidence of substantial publication bias was identified based on funnel plot inspection and statistical tests [29].

The observed findings may be partly explained by the methodological and clinical similarities among the included studies. All studies administered methylene blue intraoperatively via the intravenous route following anesthesia induction, with comparable dosing regimens. This relative homogeneity in intervention characteristics may have contributed to the stability of the pooled estimates, while also limiting the ability to explore potential sources of variability through subgroup analyses [8, 9, 29]. However, the inclusion of only three randomized controlled trials necessitates cautious interpretation, as the limited number of studies restricts statistical power and generalizability.

From a mechanistic perspective, postoperative delirium is a multifactorial condition involving complex interactions between neuroinflammation, oxidative stress, and disrupted neuronal metabolism [30–34]. Although biomarker studies have identified associations between inflammatory mediators and delirium, causal pathways remain incompletely understood [32]. Nevertheless, pharmacological interventions targeting these biological pathways—such as dexmedetomidine and sevoflurane—have been associated with improvements in cognitive and behavioral outcomes, supporting the clinical relevance of these mechanisms [34–38]. In this context, methylene blue may exert beneficial effects through its regulatory actions on neuroinflammation, oxidative stress, and mitochondrial function [39–41].

Experimental and clinical evidence suggests that methylene blue may attenuate surgery-induced neuroinflammatory responses, preserve mitochondrial function, and improve cellular energy metabolism [30, 31, 39–41]. These mechanisms overlap with key pathophysiological processes implicated in postoperative delirium, providing a plausible explanation for the observed reduction in delirium incidence. Accordingly, the findings of this meta-analysis are consistent with the hypothesis that targeting neuroinflammatory and oxidative pathways may represent a potential strategy for reducing postoperative neurocognitive complications [31, 32].

From a clinical perspective, the reduction in postoperative delirium observed in this analysis suggests that intraoperative methylene blue administration may represent a feasible pharmacological approach, particularly in patients at high risk of delirium. However, given the limited number of available studies and the relatively narrow range of surgical populations examined, further large-scale randomized controlled trials are required to determine optimal dosing, timing, and patient selection criteria. In addition, future studies should evaluate long-term cognitive outcomes and incorporate standardized delirium assessment tools to improve comparability across studies.

This study has several strengths. It is the first meta-analysis to evaluate the effects of intraoperative methylene blue administration on postoperative delirium using pooled evidence. The inclusion of randomized controlled trials, the relative methodological consistency across studies, and the use of multiple approaches to assess publication bias (funnel plot, Egger’s regression test, and Kendall’s tau) support the robustness of the findings.

### Strength and limitations

The most important limitation of this meta-analysis is the small number of included studies (*n* = 3). Although the studies were methodologically and clinically homogeneous, the generalizability of the findings derived from a limited number of trials should be interpreted with caution. The small number of studies precluded statistically meaningful subgroup analyses according to different surgical types, patient subgroups, dosing regimens, or timing of administration, thereby limiting the ability to evaluate the relative effects of methylene blue across specific clinical settings. In addition, incomplete blinding due to urine discoloration associated with methylene blue may have introduced potential bias related to deviations from intended interventions and outcome assessment. Nevertheless, no study was judged to be at high risk of bias. Finally, although the limited number of studies may have reduced the statistical power of publication bias assessments, no evidence indicating substantial publication bias was identified in the present analyses.

## Conclusion

This meta-analysis demonstrates that intraoperative administration of methylene blue significantly reduces the incidence of postoperative delirium in surgical patients. Pooled evidence from three randomized controlled trials indicates that the effect of methylene blue on postoperative delirium is consistent and that no heterogeneity was observed across studies. In addition, the available data suggest that methylene blue use does not increase the incidence of serious adverse events and appears to have an acceptable safety profile. Nevertheless, given the limited number of included studies and the similarity of intervention characteristics, the findings should be interpreted with caution. The inability to perform subgroup analyses restricts a more detailed evaluation of the relative effects of methylene blue across different surgical types, patient subgroups, and dosing regimens. Therefore, larger, multicenter randomized controlled trials encompassing diverse surgical populations are warranted. Overall, the current evidence suggests that intraoperative methylene blue administration may represent a potentially effective and safe pharmacological approach for preventing postoperative delirium. Future high-quality studies are expected to further clarify optimal dosing, timing of administration, and underlying mechanisms, thereby supporting the translation of these findings into clinical practice.

## Data Availability

The datasets used and/or analysed during the current study are available from the corresponding author on reasonable request.
